# Prognostic impact of AnxA1 and AnxA2 gene expression in triple-negative breast cancer

**DOI:** 10.18632/oncotarget.23627

**Published:** 2017-12-23

**Authors:** Lee D. Gibbs, Jamboor K. Vishwanatha

**Affiliations:** ^1^ Institute for Molecular Medicine and Texas Center for Health Disparities, University of North Texas Health Science Center, Fort Worth, TX 76017, USA

**Keywords:** annexin, triple-negative breast cancer, prognosis, survival, relapse

## Abstract

**Objective:**

Previous studies have shown Annexin A1 (AnxA1) and Annexin A2 (AnxA2) association with the aggressive behavior of Triple Negative Breast Cancer (TNBC). Our aim was to determine the correlation of AnxA1 and AnxA2 with poor prognosis of TNBC patients.

**Methods:**

We analyzed the gene expression of the human annexin family from microarray datasets and correlated with clinical outcomes to determine their ability to predict prognosis.

**Results:**

Within a mean follow-up time of 57.2 months in our TNBC cohort, high AnxA1 expression was an independent indicator of poor overall survival (OS) [hazard ratio (HR), 2.14; 95% confidence interval (CI), 1.22-3.78] and relapse-free survival (RFS) prognosis [HR, 1.66; 95% CI, 1.28-2.17]. Additionally, high AnxA2 expression was an independent indicator of poor OS [HR, 2.66; 95% CI, 1.14-6.25], RFS [HR, 1.45; 95% CI, 1.12-1.89], RFS [HR, 1.45; 95% CI, 1.12-1.89), and distant metastasis free survival (DMFS) prognosis [HR, 1.5; 95% CI, 1.16-1.95]. Analyses of TNBC patients with both high AnxA1 and AnxA2, demonstrates a significant decrease in OS (*P*=0.0017) and RFS (*P*=0.0002) when compared to the expression of genes independently. Furthermore, AnxA1 prognostic impact relies on high AnxA2 expression and both are preferential for TNBC when compared to other breast cancer subtypes.

**Conclusion:**

Together these findings indicate that AnxA1 and AnxA2 are preferential dual prognostic predictors among TNBC patients.

## INTRODUCTION

The American Cancer Society (ACS) predicts that in 2017, 252,710 new cases of Invasive breast cancer and 63,410 new cases of carcinoma *in situ* (CIS) will be diagnosed in the United Sates [1]. Further, a predicted 40,610 women will succumb to this disease. Based on a 2011-2013 report by the Surveillance, Epidemiology, and End Results (SEER) Program, approximately 12.4% of women will be diagnosed with breast cancer at some point during their lifetime [2]. The yearly statistics from all major national health related organizations such as: The National Institute for Health (NIH), National Cancer Institute (NCI), Centers for Disease Control and Prevention (CDCP), World Health Organization (WHO), SEER, ACS and others have detailed the risk of breast cancer nationally and internationally. Although, breast cancer is the second leading cause of cancer death among women, the discoveries made in research have dropped breast cancer rates significantly since 1989 [3–5]. This would suggest that continued strides in raising awareness of early detection, discovering innovative screening techniques, and establishing appropriate clinical recommendations to adequately diagnose and prognosticate this disease will continue to decrease the overall number of lives affected by breast cancer.

At a glance, we often think of breast cancer as a single homogenous disease. But, in fact it is a heterogeneous complex of diseases. This complex has been delineated into several molecular subtypes that have different treatment options, responses to therapy, and clinical outcomes. These advances in breast cancer classification have led to the identification of three molecular markers: Estrogen Receptor (ER), Progesterone Receptor (PR), and Human Epidermal Growth Factor Receptor (HER2) [6–9]. These analyses separated breast cancer into three subtypes: ER+ and/or PR+, HER2+, and Triple-Negative (lacks expression of all three markers) breast cancer subtype. The presentation of these markers is determined after breast lumpectomy and sent to a histology lab where a pathologist performs immunohistochemistry or *in situ* hybridization to determine the expression of these markers. Taken together, tumor size, tumor grade, and nodal involvement are conventionally used for prognosis and therapeutic management of a patient's disease [10–12]. Unfortunately, some patients do not benefit from this standard of care and often have higher risk of recurrence, distant metastasis, and death.

The development of microarrays and genomic sequencing has expanded our Interpretation of breast cancer classification [13, 14]. Sorlie et al. utilized gene expression profiling (GEP) to create a distinctive molecular portrait of breast cancer using 456 cDNA clones, and reclassified tumors into five intrinsic clinical subtypes: Luminal A (ER+ and/or PR+/HER2-), Luminal B (ER+ and/or PR+/HER2+), Basal-like (ER- and/or PR-/HER2-), HER2+ (ER- and/or PR-/HER2+), and normal-like tumors [15, 16]. These analyses also revealed that the reclassification of these subtypes could also potentiate clinical outcomes and prognoses [17]. Luminal A tumors have the more favorable prognoses and make up approximately 40% of all breast cancer cases. It is often diagnosed at lower grades (well differentiation of cells) and their morphology mimics the luminal epithelial component of the breast [15, 18]. Patients with Luminal A tumors are often given targeted endocrine therapies toward the expression of their receptors such as anti-estrogen or aromatase inhibitors [19]. Luminal B tumors are very similar to Luminal A tumors as they express the ER receptor and have favorable prognoses [20]. Additionally, this subtype expresses HER2 receptor and has higher expression of proliferative genes in comparison to Luminal A [18]. These tumors make up approximately 20% of breast cancer cases and tend to be diagnosed at higher tumor grades than Luminal A tumors. HER2 is as a unique identifier of a subset of breast cancer patients that was found after the discoveries of ER and PR. Unlike, ER and PR, HER2 can be identified by immunohistochemistry (IHC) and fluorescence *in situ* hybridization (FISH). Although these two experimental techniques have been perfected throughout their use, all HER2 positive tumors do not show expression at the protein and transcriptional level. Thus, HER2 classification is also characterized by expression of other genes in the HER2 amplicon such as: Growth Factor Receptor Bound Protein 7 (GRB7), Post-GPI Attachment To Proteins 3 (PGAP3), and TP53 (Tumor Protein 53) mutation [16, 21]. HER2 tumors are often aggressive and have poor prognoses. They are more likely to be diagnosed at higher grades and are usually treated by a well-known targeted therapy, Trastuzumb (HER2 antibody), coupled with radiation. Additionally, these tumors are sensitive to anthracycline and taxane-based neoadjuvant chemotherapy [22]. Triple-Negative breast cancer (TNBC) makes up approximately 15-20% of breast cancer diagnoses and consists of 60-90% basal like tumors, mimicking basal epithelial cells found in other parts of the body [16, 23]. TNBC has a high proliferative index and has high expression of basal markers (such as keratins 5, 6, 14, 17, Epidermal Growth Factor Receptor) [16, 23]. They are often presented as higher grade (poor differentiation of cells) and have the worst prognosis [24]. TNBC is the most aggressive breast cancer subtype and is unresponsive to anti-hormonal and Her2-targeted therapies due to the absence of hormone receptors and Her2 expression. Similar to other aggressive breast cancers, TNBC tumors respond best to a combination of chemotherapy and radiation. Though characterization of these entities of breast cancer have advanced our understanding of clinical outcomes and therapeutic approaches, we must continue studying tumor heterogeneity to yield the best descriptive analysis of each patient's tumor.

Annexins were first identified in 1977 as intracellular proteins that were associated with intracellular membranes [25]. Annexins are a family of calcium dependent phospholipid binding proteins that contain a conserved structural element, the “annexin repeat”, a segment of approximately seventy amino acid residues located in its carboxyl-terminus, and a divergent amino-terminus [26]. The annexin family consists of 12 members (AnxA1–A13, AnxA12 is unassigned) that make up the human annexin family [26]. Annexins have been investigated more than fifteen years to study their relationship with breast cancer. These studies have demonstrated that certain annexins are associated with proliferation, migration, invasion, angiogenesis and metastasis. Throughout the years of investigation Annexin A1 (AnxA1), Annexin A2 (AnxA2), Annexin A3 (AnxA3), Annexin A4 (AnxA4), Annexin A5 (AnxA5), Annexin A6 (AnxA6), and Annexin A8 (AnxA8) have all been identified as potential modulators of breast cancer progression. Evidence has shown AnxA1, AnxA2, AnxA8 are associated with the basal-like phenotype and potentiates poor prognosis of basal-like breast cancer [27, 28, 29]. Recent studies have shown AnxA3 potential as a serum biomarker and regulator of apoptosis [30]. AnxA4 and AnxA5 are expressed in breast cancer tissues and upregulation of AnxA4 promotes chemo-resistance of breast cancer [31, 32, 33, 34]. AnxA6 expression is reduced in breast cancer cells and when expressed terminates EGFR signaling [35]. AnxA2, the annexin protein that has been studied in detail in breast cancer, has been shown to promote TNBC progression, through angiogenesis and metastasis [36, 37, 38].

## RESULTS

### AnxA1, AnxA2, and AnxA6 are associated with TNBC and are associated with poor clinical outcomes

**Figure 1:**
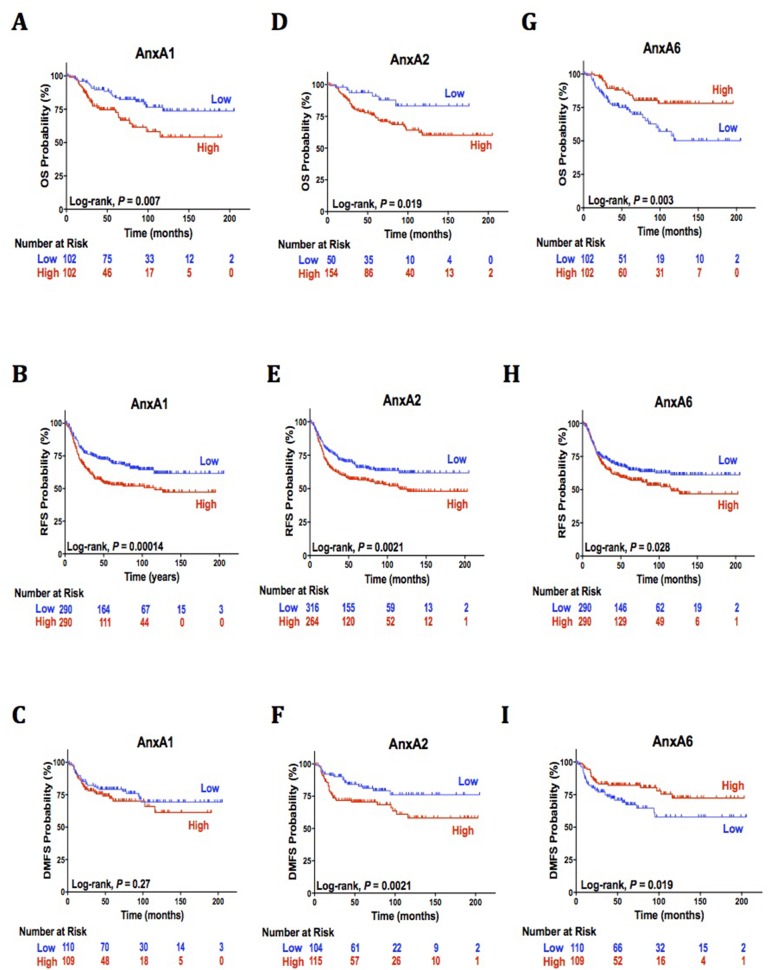
AnxA1, AnxA2, and AnxA6 independent association with clinical outcomes **(A-I)** (A) Kaplan-Meier curves with univariate analyses (log-rank) for patients with low AnxA1 gene expression versus high AnxA1 expression from tumors in triple negative breast cancer for overall survival (B) relapse free survival and (C) distant metastasis free survival. (D) Kaplan-Meier curves with univariate analyses (log-rank) for patients with low AnxA2 gene expression versus high AnxA2 expression from tumors in triple negative breast cancer for overall survival (E) relapse free survival and (F) distant metastasis free survival. (G) Kaplan-Meier curves with univariate analyses (log-rank) for patients with low AnxA2 gene expression versus high AnxA2 expression from tumors in triple negative breast cancer for overall survival (H) relapse free survival and (I) distant metastasis free survival.

In our TNBC cohort (mean observation time = 57.2 months, median = 45.5 months), 51 deaths of any cause, 220 reoccurrences, and 56 metastatic events were reported. All annexins gene expression was analyzed to determine their individual association with TNBC (not shown). AnxA1, AnxA2, and AnxA6 were the only annexins identified to be significantly associated with clinical outcomes of TNBC patients in comparison with all other breast cancer subtypes (Table 1). Significantly worse OS (*P* = 0.007, Figure 1A) and RFS (*P* < 0.0001 Figure 1B) was observed among patients with high AnxA1 expression compared to low expression and is independently associated with poor OS prognosis [hazard ratio (HR), 2.14; 95% (CI), 1.22-3.78, Table 1] and poor RFS prognosis [HR, 1.66; 95% CI, 1.28-2.17, Table 1]. High AnxA1 expression was not significantly associated with DMFS or poor prognosis [*P* < 0.27, HR, 1.33; 95% CI, 0.79-2.24, Table 1, Figure 1C]. Similar to AnxA1, AnxA2 is associated with unfavorable clinical outcomes and poor prognosis. Significantly worse OS, RFS, and DMFS (*P* =0.019, *P* = 0.0051, *P* = 0.0021, Figure 1D, 1E, 1F, respectively) were observed among patients with high AnxA2 expression compared to low expression. High AnxA2 is independently associated with poor OS [HR, 2.14; 95% CI, 1.22-3.78, Table 1], RFS [HR, 1.45; 95% CI, 1.12-1.89, Table 1], and poor DMFS prognosis [HR, 1.5; 95% CI, 1.16-1.95, Table 1]. AnxA6 analysis shows conflicting results as high AnxA6 expression significantly correlated to unfavorable (RFS, *P* < 0.028, Figure 1H) and favorable prognosis (OS, *P* = 0.003, Figure 1G; DMFS, *P* = 0.019, Figure 1I). These results could not determine AnxA6 as a potential reliable prognostic predictor.

**Table 1:** Survival analysis of AnxA1 and AnxA2 with clinical outcomes in patients with breast cancer (intrinsic subtypes)

Variables	Overall Survival		Relapse Free Survival		Distant Metastasis Free Survival	
	HR *^a^*(95% CI)*^b^*	P-value*^c^*	HR*^a^* (95% CI)*^b^*	P-value*^c^*	HR *^a^*(95% CI)*^b^*	P-value*^c^*
**All Breast Cancer Subtypes**						
AnxA1 (high vs. low)	1.02 (0.8-1.29)	0.89	1.07 (0.96-1.2)	0.22	0.95 (0.78-1.16)	0.62
AnxA2 (high vs. low)	1.04 (0.82-1.32)	0.74	1.11 (0.99-1.25)	0.067	1.05 (0.86-1.29)	0.63
AnxA6 (high vs. low)	0.8 (0.63-1.02)	0.067	0.96 (0.86-1.08)	0.52	0.87 (0.71-1.07)	0.18
**Luminal A Breast Cancer**						
AnxA1 (high vs. low)	0.93 (0.64-1.36)	0.7	0.95 (0.8-1.14)	0.58	0.92 (0.69-1.24)	0.58
AnxA2 (high vs. low)	0.71 (0.48-1.04)	0.08	0.94 (0.79-1.12)	0.48	1 (0.74-1.35)	1
AnxA6 (high vs. low)	1.19 (0.81-1.73)	0.38	0.89 (0.75-1.06)	0.2	1.07 (0.79-1.44)	0.66
**Luminal B Breast Cancer**						
AnxA1 (high vs. low)	0.86 (0.57-1.31)	0.49	0.92 (0.75-1.12)	0.41	0.78 (0.53-1.13)	0.19
AnxA2 (high vs. low)	1.33 (0.88-2.01)	0.18	1.12 (0.91-1.37)	0.28	1.05 (0.72-1.52)	0.81
AnxA6 (high vs. low)	0.85 (0.56-1.28)	0.43	1.07 (0.87-1.31)	0.52	0.76 (0.52-1.11)	0.16
**HER2+ Breast Cancer**						
AnxA1 (high vs. low)	0.61 (0.28-1.33)	0.21	0.72 (0.47-1.09)	0.12	1.36 (0.71-2.59)	0.35
AnxA2 (high vs. low)	0.77 (0.36-1.65)	0.5	1.01 (0.66-1.52)	0.98	1.27 (0.66-2.42)	0.47
AnxA6 (high vs. low)	1.36 (0.63-2.94)	0.43	1.41 (0.93-2.15)	0.11	1.41 (0.74-2.71)	0.3
**Triple-negative/Basal Breast Cancer**						
AnxA1 (high vs. low)^*^	**2.14 (1.22-3.78)**	**0.007**	**1.66 (1.28-2.17)**	**0.00014**	**1.33 (0.79-2.24)**	**0.27**
AnxA2 (high vs. low)^*^	**2.66 (1.14-6.25)**	**0.019**	**1.45 (1.12-1.89)**	**0.0051**	**1.5 (1.16-1.95)**	**0.0021**
AnxA6 (high vs. low)^*^	**0.43 (0.24-0.76)**	**0.003**	**1.34(1.03-1.74)**	**0.028**	**0.54 (0.32-0.91)**	**0.019**

*^a^* HR=Hazard Ratio.

*^b^* CI= Confidence Interval.

*^c^ P*-value = *P* < 0.05 considered significant.

^*^ = Significant genes (Genes and subtypes in bold to indicate preference for subtype).

### AnxA1 and AnxA2 have dual association with TNBC and poor clinical outcomes

**Figure 2:**
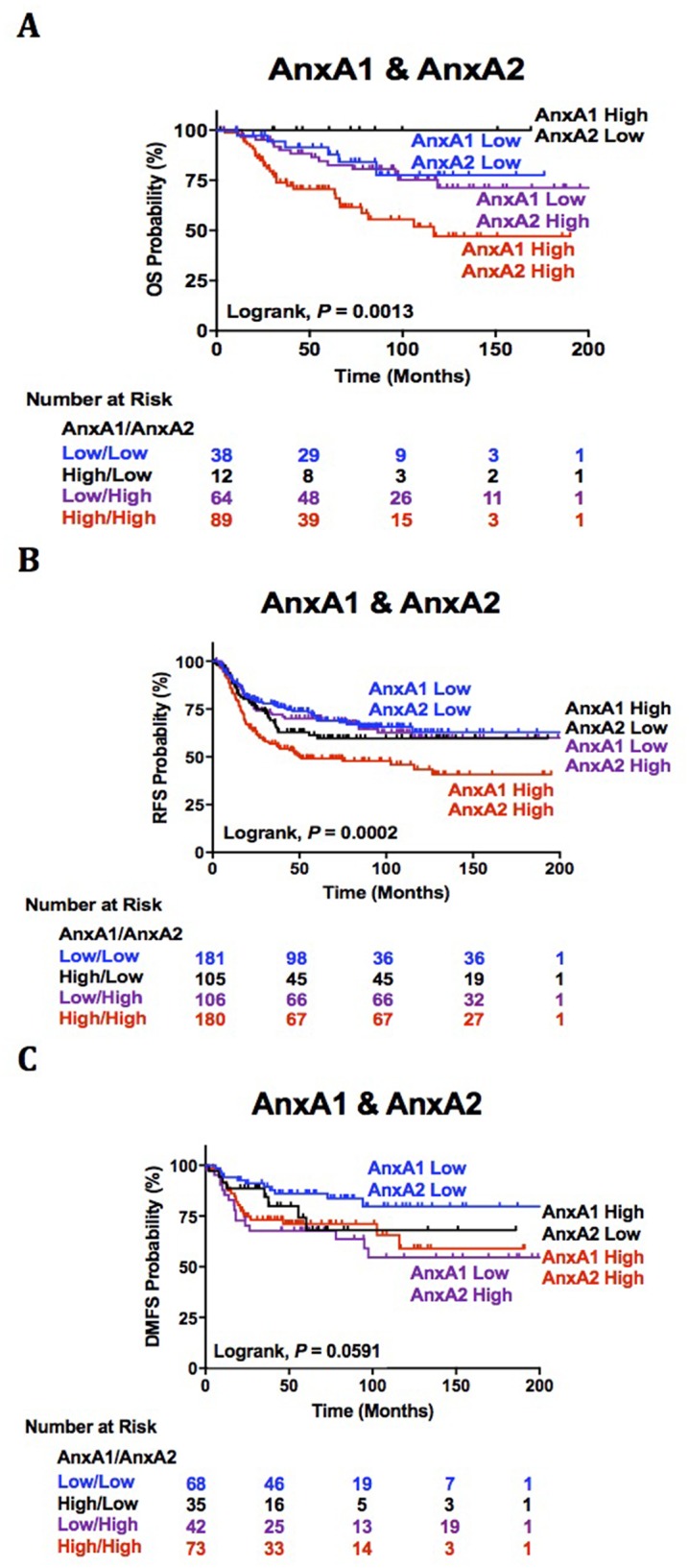
AnxA1 and AnxA2 dual association with clinical outcomes **(A-C)** Survival estimations of TNBC patients stratified by combined tumor AnxA1 and AnxA2 gene expression status are shown for (A) overall survival (B), relapse free survival (C) and distant metastasis free survival.

Our analysis of AnxA1 and AnxA2 dual association with clinical outcomes reveals extremely poor OS and RFS in TNBC patients with high AnxA1/AnxA2 expression. High AnxA1/low AnxA2 and low AnxA1/low AnxA2 expression has the most favorable OS and RFS respectively (*P* = 0.0013, Figure 2A; *P* = 0.0002, Figure 2B). Although our analysis of AnxA1 and AnxA2 dual association with DMFS was not significant (P = 0.0591, Figure 2C), we observed an interesting trend of unfavorable DMFS in patients with low AnxA1/high AnxA2 and a more favorable outcome in patients with low AnxA1/low AnxA2. Thus, our evidence suggests AnxA1 prognostic prediction power of decreased survival and high recurrence relies on high AnxA2 expression.

## DISCUSSION

Previous studies striving for more reliable predictors of TNBC progression focus on immunohistochemical analysis and expression profiles [6, 7, 39–42]. However, none of the proposals have been implemented as clinical recommendations to adequately determine prognosis. The data here comprehensively demonstrates that AnxA1 and AnxA2 may be translated into novel markers of prognostic power. Application of such markers will assist in overcoming the current limitations of histological classification and prognostic evaluation.

Information on AnxA1 and AnxA2 gene expression may allow the clinician to identify a patients’ diseases at higher risk than patients’ that may have favorable outcomes. Additionally, our lab's previous discovery of the reciprocal relationship between HER-2 and AnxA2 supports the correlation observed between high AnxA2 and poor clinical outcomes in TNBC [43]. Further, our recent study of the functional role of AnxA2 in establishing a favorable tumor microenvironment for migrating TNBC cells provides additional support for AnxA2 as an independent and reliable prognostic predictor for DMFS [44]. Interestingly, our results demonstrate AnxA1 prognostic predictive power is driven by high expression of AnxA2 and significantly increases a patient risk for death and relapse. Although AnxA6 expression had significant correlation with clinical outcomes of TNBC, ambiguous results and lack of supporting literature on its role in TNBC did not warrant further investigation.

Although this study was informative, the present study had several limitations. First, the retrospective nature of this study should be noted. To decrease potential biases, however, we analyzed large numbers of patients from multiple institutions and several investigators. Further, the number of cases and lack of detailed clinical information does not allow for robust biological conclusions on the effect of age, menopause status, stage, tumor grade, and race/ethnicity to adequately assess the association of AnxA1 and AnxA2 with the disparity of TNBC in pre-menopausal and women of African descent [15]. In conclusion, AnxA1 and AnxA2 are dually associated with unfavorable clinical outcomes and may be useful tools in predicting poor prognosis in TNBC patients.

## MATERIALS AND METHODS

### Databases and analyses

Survival data of all members of the human annexin family were derived from information in the Kaplan-Meier Plotter (http://kmplot.com/analysis/) consisting of compiled results from microarray datasets of 5, 143 breast tissue samples. Gene expression of solid breast tumors was obtained from combining 23 Gene Expression Omnibus (GEO) (https://www.ncbi.nlm.nih.gov/geo/) microarray datasets from Affymetrix HG-U133A, HG-U133 Plus 2.0 and HG-U133A 2.0 [45]. The cutoff values for AnxA1 and AnxA2 expression for “low” and “high” were determined using the median of their individual gene expression range. Overall Survival (OS) was defined as the interval between the date of surgery and date of death from any cause or last contact. Relapse Free Survival (RFS) was defined as the interval from the date of surgery to the date of recurrence diagnosis or last contact. Distant Metastasis Free Survival (DMFS) was defined as the interval from the date of surgery to the date of metastasis diagnosis to brain, lungs, bone or last contact. Graphpad Prism 7 was utilized for our survival analyses. Survival analyses were based on Kaplan–Meier estimations and the log-rank test was used to analyze differences in survival durations in which the assumption of the test is that of proportional hazards (reported at 95% Confidence Interval (CI)). These analyses determined the impacts of the annexins on OS, RFS, and DMFS. All statistical tests were two-sided, and *P* values <0.05 were considered statistically significant.
